# The Alkaline Diet: Is There Evidence That an Alkaline pH Diet Benefits Health?

**DOI:** 10.1155/2012/727630

**Published:** 2011-10-12

**Authors:** Gerry K. Schwalfenberg

**Affiliations:** University of Alberta, Suite No. 301, 9509-156 Street, Edmonton, AB, Canada T5P 4J5

## Abstract

This review looks at the role of an alkaline diet in health. Pubmed was searched looking for articles on pH, potential renal acid loads, bone health, muscle, growth hormone, back pain, vitamin D and chemotherapy. Many books written in the lay literature on the alkaline diet were also reviewed and evaluated in light of the published medical literature. There may be some value in considering an alkaline diet in reducing morbidity and mortality from chronic diseases and further studies are warranted in this area of medicine.

## 1. Background

Life on earth depends on appropriate pH levels in and around living organisms and cells. Human life requires a tightly controlled pH level in the serum of about 7.4 (a slightly alkaline range of 7.35 to 7.45) to survive [1]. 

As a comparison, in the past 100 years with increasing industrialization, the pH of the ocean has dropped from 8.2 to 8.1 because of increasing CO_2_ deposition. This has a negative impact on life in the ocean [1, 2] and may lead to the collapse of the coral reefs [3]. Even the pH of the soil in which plants are grown can have considerable influence on the mineral content of the food we eat (as minerals are used as buffers to maintain pH). The ideal pH of soil for the best overall availability of essential nutrients is between 6 and 7. Acidic soils below pH of 6 may have reduced calcium and magnesium, and soil above pH 7 may result in chemically unavailable iron, manganese, copper and zinc. Adding dolomite and manure are ways of raising pH in an acid soil environment when the pH is below 6 [4].

When it comes to the pH and net acid load in the human diet, there has been considerable change from the hunter gather civilization to the present [5]. With the agricultural revolution (last 10,000 years) and even more recently with industrialization (last 200 years), there has been an decrease in potassium (K) compared to sodium (Na) and an increase in chloride compared to bicarbonate found in the diet [6]. The ratio of potassium to sodium has reversed, K/Na previously was 10 to 1 whereas the modern diet has a ratio of 1 to 3 [7]. It is generally accepted that agricultural humans today have a diet poor in magnesium and potassium as well as fiber and rich in saturated fat, simple sugars, sodium, and chloride as compared to the preagricultural period [6]. This results in a diet that may induce metabolic acidosis which is mismatched to the genetically determined nutritional requirements [8]. With aging, there is a gradual loss of renal acid-base regulatory function and a resultant increase in diet-induced metabolic acidosis while on the modern diet [9]. A low-carbohydrate high-protein diet with its increased acid load results in very little change in blood chemistry, and pH, but results in many changes in urinary chemistry. Urinary magnesium levels, urinary citrate and pH are decreased, urinary calcium, undissociated uric acid, and phosphate are increased. All of these result in an increased risk for kidney stones [10]. 

Much has been written in the lay literature as well as many online sites expounding on the benefits of the alkaline diet. This paper is an attempt to balance the evidence that is found in the scientific literature.

## 2. The Role of pH in Various Cells, Organs, and Membranes

The pH in our body may vary considerably from one area to another with the highest acidity in the stomach (pH of 1.35 to 3.5) to aid in digestion and protect against opportunistic microbial organisms. But even in the stomach, the layer just outside the epithelium is quite basic to prevent mucosal injury. It has been suggested that decreased gastric lining secretion of bicarbonates and a decrease in the alkaline/acid secretion in duodenal ulcer patients may play a significant role in duodenal ulcers [11]. The skin is quite acidic (pH 4–6.5) to provide an acid mantle as a protective barrier to the environment against microbial overgrowth. There is a gradient from the outer horny layer (pH 4) to the basal layer (pH 6.9) [12]. This is also seen in the vagina where a pH of less than 4.7 protects against microbial overgrowth [13]. 

The urine may have a variable pH from acid to alkaline depending on the need for balancing the internal environment. Acid excretion in the urine can be estimated by a formula described by Remer (sulfate + chloride + 1.8x phosphate + organic acids) minus (sodium + potassium + 2x calcium + 2x magnesium) mEq [14]. Foods can be categorized by the potential renal acid loads (PRALs) see Table 2. Fruits, vegetables, fruit juices, potatoes, and alkali-rich and low phosphorus beverages (red and white wine, mineral soda waters) having a negative acid load. Whereas, grain products, meats, dairy products, fish, and alkali poor and low phosphorus beverages (e.g., pale beers, cocoa) have relatively high acid loads [15]. Measurement of pH of the urine (reviewed in a recent study with two morning specimens done over a five-year span) did not predict bone fractures or loss of bone mineral density [16]. However, this may not be reflective of being on an alkaline or acid diet throughout this time. For more details, see Table 1.

## 3. Chronic Acidosis and Bone Disease

Calcium in the form of phosphates and carbonates represents a large reservoir of base in our body. In response to an acid load such as the modern diet these salts are released into the systemic circulation to bring about pH homeostasis [7]. It has been estimated that the quantity of calcium lost in the urine with the modern diet over time could be as high as almost 480 gm over 20 years or almost half the skeletal mass of calcium [21]. However, urinary losses of calcium are not a direct measure of osteoporosis. There are many regulatory factors that may compensate for the urinary calcium loss. When the arterial pH is in the normal range, a mild reduction of plasma bicarbonate results in a negative calcium balance which could benefit from supplementing bicarbonate in the form of potassium bicarbonate [22]. It has been found that bicarbonate, which increases the alkali content of a diet, but not potassium may attenuate bone loss in healthy older adults [23]. The bone minerals that are wasted in the urine may not have complete compensation through intestinal absorption, which is thought to result in osteoporosis. However, adequate vitamin D with a 25(OH)D level of >80 nmol/L may allow for appropriate intestinal absorption of calcium and magnesium and phosphate when needed [24]. Sadly, most populations are generally deficient in vitamin D especially in northern climates [25]. In chronic renal failure, correction of metabolic acidosis with bicarbonate significantly improves parathyroid levels and levels of the active form of vitamin D 1,25(OH)2D_3_ [26]. Recently, a study has shown the importance of phosphate in Remer's PRAL formula. According to the formula it would be expected that an increase in phosphate should result in an increase in urinary calcium loss and a negative calcium balance in bone [27]. It should be noted that supplementation with phosphate in patients with bed rest reduced urinary calcium excretion but did not prevent bone loss [28]. The most recent systematic review and meta-analysis has shown that calcium balance is maintained and improved with phosphate which is quite contrary to the acid-ash hypothesis [29]. As well a recent study looking at soda intake (which has a significant amount of phosphate) and osteoporosis in postmenopausal American first nations women did not find a correlation [30]. It is quite possible that the high acid content according to Remer's classification needs to be looked at again in light of compensatory phosphate intake. There is online information promoting an alkaline diet for bone health as well as a number of books. However, a recent systematic review of the literature looking for evidence supporting the alkaline diet for bone health found no protective role of dietary acid load in osteoporosis [31]. 

 Another element of the modern diet is the excess of sodium in the diet. There is evidence that in healthy humans the increased sodium in the diet can predict the degree of hyperchloremic metabolic acidosis when consuming a net acid producing diet [32]. As well, there is evidence that there are adverse effects of sodium chloride in the aging population. A high sodium diet will exacerbate disuse-induced bone and muscle loss during immobilization by increasing bone resorption and protein wasting [33]. Excess dietary sodium has been shown to result in hypertension and osteoporosis in women [34, 35]. As well, dietary potassium which is lacking in the modern diet would modulate pressor and hypercalciuric effects of excess of sodium chloride [36].

Excess dietary protein with high acid renal load may decrease bone density if not buffered by ingestion of supplements or foods that are alkali rich [37]. However, adequate protein is necessary for prevention of osteoporosis and sarcopenia; therefore, increasing the amount of fruit and vegetables may be necessary rather than reducing protein [38].

## 4. Alkaline Diets and Muscle

As we age, there is a loss of muscle mass, which may predispose to falls and fractures. A three-year study looking at a diet rich in potassium, such as fruits and vegetables, as well as a reduced acid load, resulted in preservation of muscle mass in older men and women [39]. Conditions such as chronic renal failure that result in chronic metabolic acidosis result in accelerated breakdown in skeletal muscle [40]. Correction of acidosis may preserve muscle mass in conditions where muscle wasting is common such as diabetic ketosis, trauma, sepsis, chronic obstructive lung disease, and renal failure [41]. In situations that result in acute acidosis, supplementing younger patients with sodium bicarbonate prior to exhaustive exercise resulted in significantly less acidosis in the blood than those that were not supplemented with sodium bicarbonate [42].

## 5. Alkaline Supplementation and Growth Hormone

It has long been known that severe forms of metabolic acidosis in children, such as renal tubular acidosis, are associated with low levels of growth hormone with resultant short stature. Correction of the acidosis with bicarbonate [7] or potassium citrate [43] increases growth hormone significantly and improved growth. The use of enough potassium bicarbonate in the diet to neutralize the daily net acid load in postmenopausal women resulted in a significant increase in growth hormone and resultant osteocalcin [44]. Improving growth hormone levels may improve quality of life, reduce cardiovascular risk factors, improve body composition, and even improve memory and cognition [45]. As well this results in a reduction of urinary calcium loss equivalent to 5% of bone calcium content over a period of 3 years [46].

## 6. Alkaline Diet and Back Pain

There is some evidence that chronic low back pain improves with the supplementation of alkaline minerals [47]. With supplementation there was a slight but significant increase in blood pH and intracellular magnesium. Ensuring that there is enough intracellular magnesium allows for the proper function of enzyme systems and also allows for activation of vitamin D [48]. This in turn has been shown to improve back pain [49].

## 7. Alkalinity and Chemotherapy

The effectiveness of chemotherapeutic agents is markedly influenced by pH. Numerous agents such as epirubicin and adriamycin require an alkaline media to be more effective. Others, such as cisplatin, mitomycin C, and thiotepa, are more cytotoxic in an acid media [50]. Cell death correlates with acidosis and intracellular pH shifts higher (more alkaline) after chemotherapy may reflect response to chemotherapy [51]. It has been suggested that inducing metabolic alkalosis may be useful in enhancing some treatment regimes by using sodium bicarbonate, carbicab, and furosemide [52]. Extracellular alkalinization by using bicarbonate may result in improvements in therapeutic effectiveness [53]. There is no scientific literature establishing the benefit of an alkaline diet for the prevention of cancer at this time.

## 8. Discussion

The human body has an amazing ability to maintain a steady pH in the blood with the main compensatory mechanisms being renal and respiratory. Many of the membranes in our body require an acid pH to protect us and to help us digest food. It has been suggested that an alkaline diet may prevent a number of diseases and result in significant health benefits. Looking at the above discussion on bone health alone, certain aspects have doubtful benefit. There does not seem to be enough evidence that milk or cheese may be as detrimental as Remer's formula suggests since phosphate does benefit bone health and result in a positive calcium balance. However, another mechanism for the alkaline diet to benefit bone health may be the increase in growth hormone and resultant increase in osteocalcin. There is some evidence that the K/Na ratio does matter and that the significant amount of salt in our diet is detrimental. Even some governments are demanding that the food industry reduce the salt load in our diet. High-protein diets may also affect bone health but some protein is also needed for good bone health. Muscle wasting however seems to be reduced with an alkaline diet and back pain may benefit from this as well. An alkaline environment may improve the efficacy of some chemotherapy agents but not others.

## 9. Conclusion

 Alkaline diets result in a more alkaline urine pH and may result in reduced calcium in the urine, however, as seen in some recent reports, this may not reflect total calcium balance because of other buffers such as phosphate. There is no substantial evidence that this improves bone health or protects from osteoporosis. However, alkaline diets may result in a number of health benefits as outlined below

1. Increased fruits and vegetables in an alkaline diet would improve the K/Na ratio and may benefit bone health, reduce muscle wasting, as well as mitigate other chronic diseases such as hypertension and strokes.
2. The resultant increase in growth hormone with an alkaline diet may improve many outcomes from cardiovascular health to memory and cognition.
3. An increase in intracellular magnesium, which is required for the function of many enzyme systems, is another added benefit of the alkaline diet. Available magnesium, which is required to activate vitamin D, would result in numerous added benefits in the vitamin D apocrine/exocrine systems.
4. Alkalinity may result in added benefit for some chemotherapeutic agents that require a higher pH.

From the evidence outlined above, it would be prudent to consider an alkaline diet to reduce morbidity and mortality of chronic disease that are plaguing our aging population. One of the first considerations in an alkaline diet, which includes more fruits and vegetables, is to know what type of soil they were grown in since this may significantly influence the mineral content. At this time, there are limited scientific studies in this area, and many more studies are indicated in regards to muscle effects, growth hormone, and interaction with vitamin D.

## Figures and Tables

**Table 1:** Ph of selected fluids, organs, and membranes.

Organ, fluid or membrane	pH	Function of pH
(1) Skin	Natural pH is between 4 and 6.5 [17]	Barrier protection from microbes
(2) Urine	4.6 to 8.0 [18]	Limit overgrowth of microbes
(3) Gastric	1.35 to 3.5	Break down protein
(4) Bile	7.6 to 8.8	Neutralize stomach acid, aid in digestion
(5) Pancreatic fluid	8.8	Neutralize stomach acid, aid in digestion
(6) Vaginal fluid	<4.7 [13]	Limit overgrowth of opportunistic microbes
(7) Cerebrospinal fluid	7.3	Bathes the exterior of the brain
(8) Intracellular fluid	6.0–7.2 [19]	Due to acid production in cells
(9) Serum venous	7.35	Tightly regulated
(10) Serum arterial	7.4	Tightly regulated

**Table 2:** Potential renal acid loads (PRALs) of selected foods [20].

Food or food group	PRAL mEq of: Cl + P0_4_ + SO_4_ − Na − K − Ca − Mg
Dairy	
Parmesan cheese	34.2
Processed cheese plain	28.7
Cheddar reduced fat	26.4
Hard cheese (average)	19.2
Fresh cheese (quark)	11.3
Cottage cheese plain	8.7
Yogurt whole milk	1.5
Ice Cream	0.8
Whole milk	0.7
Buttermilk	0.5

Eggs	
Eggs yolk	23.4
Eggs white	1.1
Eggs chicken whole	8.2

Meats	
Corned beef	13.2
Luncheon meat canned	10.2
Turkey	9.9
Veal	9.0
Lean beef	7.8
Frankfurters	6.7

Sugars	
Sugar white	−0.1
Honey	−0.3

Vegetables	
Cucumber	−0.8
Broccoli	−1.2
Tomato	−3.1
Eggplant	−3.4
Celery	−5.2
Spinach	−14.0

Fats and Oils	
Butter	0.6
Margarine	−0.5
Olive oil	0.0

Fruits and nuts and fruit juices	
Peanuts	8.3
Walnuts	6.8
Grape juice unsweetened	−1.0
Orange juice unsweetened	−2.9
Apples or apple juice unsweetened	−2.2
Apricots	−4.8
Banana	−5.5
Black currents	−6.5
Raisins	−21.0

Grains and grain products	
Brown Rice	12.5
Rolled Oats	10.7
Spaghetti whole meal	7.3
Spaghetti white	6.5
Cornflakes	6.0
Rice white	4.6
Bread rye flower	4.1
Bread whole wheat	1.8

Legumes	
Lentils green and brown	3.5
Green beans	−3.1

Fish	
Trout brown	10.8
Cod fillets	7.1

Beverages	
Beer pale	0.9
Coca-Cola	0.4
Beer draft	−0.2
Wine white	−1.2
Coffee infusion	−1.4
Wine red	−2.4

## References

[1] Waugh A, Grant A (2007). *Anatomy and Physiology in Health and Illness*.

[2] University, Birmingham oAa Oceans reveal further impacts of climate change.

[3] Hoegh-Guldberg O, Mumby PJ, Hooten AJ (2007). Coral reefs under rapid climate change and ocean acidification. *Science*.

[4] Dam-ampai SO J, Nilnond C (2005). Effect of cattle manure and dolomite on soil properties and plant growth in acid upland soils. *Songklanakarin Journal of Science and Technologh*.

[5] Ströhle A, Hahn A, Sebastian A (2010). Estimation of the diet-dependent net acid load in 229 worldwide historically studied hunter-gatherer societies. *American Journal of Clinical Nutrition*.

[6] Sebastian A, Frassetto LA, Sellmeyer DE, Merriam RL, Morris RC (2002). Estimation of the net acid load of the diet of ancestral preagricultural Homo sapiens and their hominid ancestors. *American Journal of Clinical Nutrition*.

[7] Frassetto L, Morris, Jr. R.C. RC, Sellmeyer DE, Todd K, Sebastian A (2001). Diet, evolution and aging—the pathophysiologic effects of the post-agricultural inversion of the potassium-to-sodium and base-to-chloride ratios in the human diet. *European Journal of Nutrition*.

[8] Konner M, Boyd Eaton S (2010). Paleolithic nutrition: twenty-five years later. *Nutrition in Clinical Practice*.

[9] Lindeman RD, Goldman R (1986). Anatomic and physiologic age changes in the kidney. *Experimental Gerontology*.

[10] Reddy ST, Wang CY, Sakhaee K, Brinkley L, Pak CY (2002). Effect of low-carbohydrate high-protein diets on acid-base balance, stone-forming propensity, and calcium metabolism. *American Journal of Kidney Diseases*.

[11] Malov YS, Kulikov AN (1998). Bicarbonate deficiency and duodenal ulcer. *Terapevticheskii Arkhiv*.

[12] Ohman H, Vahlquist A (1994). In vivo studies concerning a pH gradient in human stratum corneum and upper epidermis. *Acta Dermato-Venereologica*.

[13] Ferris DG, Francis SL, Dickman ED, Miler-Miles K, Waller JL, McClendon N (2006). Variability of vaginal pH determination by patients and clinicians. *Journal of the American Board of Family Medicine*.

[14] Remer T, Manz F (1994). Estimation of the renal net acid excretion by adults consuming diets containing variable amounts of protein. *American Journal of Clinical Nutrition*.

[15] Remer T (2000). Influence of diet on acid-base balance. *Seminars in Dialysis*.

[16] Fenton TR, Eliasziw M, Tough SC, Lyon AW, Brown JP, Hanley DA (2010). Low urine pH and acid excretion do not predict bone fractures or the loss of bone mineral density: a prospective cohort study. *BMC Musculoskeletal Disorders*.

[17] Boelsma E, van de Vijver LPL, Goldbohm RA, Klöpping-Ketelaars IAA, Hendriks HFJ, Roza L (2003). Human skin condition and its associations with nutrient concentrations in serum and diet. *American Journal of Clinical Nutrition*.

[18] Ince BA, Anderson EJ, Neer RM (2004). Lowering dietary protein to U.S. recommended dietary allowance levels reduces urinary calcium excretion and bone resorption in young women. *Journal of Clinical Endocrinology and Metabolism*.

[19] Boron WF (2004). Regulation of intracellular pH. *Advances in Physiology Education*.

[20] Remer T, Manz F (1995). Potential renal acid load of foods and its influence on urine pH. *Journal of the American Dietetic Association*.

[21] Fenton TR, Eliasziw M, Lyon AW, Tough SC, Hanley DA (2008). Meta-analysis of the quantity of calcium excretion associated with the net acid excretion of the modern diet under the acid-ash diet hypothesis. *American Journal of Clinical Nutrition*.

[22] Sebastian A, Morris RC (1994). Improved mineral balance and skeletal metabolism in postmenopausal women treated with potassium bicarbonate. *New England Journal of Medicine*.

[23] Dawson-Hughes B, Harris SS, Palermo NJ, Castaneda-Sceppa C, Rasmussen HM, Dallal GE (2009). Treatment with potassium bicarbonate lowers calcium excretion and bone resorption in older men and women. *Journal of Clinical Endocrinology and Metabolism*.

[24] Heaney RP, Dowell MS, Hale CA, Bendich A (2003). Calcium absorption varies within the reference range for serum 25-hydroxyvitamin D. *Journal of the American College of Nutrition*.

[25] Schwalfenberg GK, Genuis SJ, Hiltz MN (2010). Addressing vitamin D deficiency in Canada: a public health innovation whose time has come. *Public Health*.

[26] Lu KC, Lin SH, Yu FC, Chyr SH, Shieh SD (1995). Influence of metabolic acidosis on serum 1,25(OH)2D3 levels in chronic renal failure. *Mineral and Electrolyte Metabolism*.

[27] Fenton TR, Lyon AW, Eliasziw M, Tough SC, Hanley DA (2009). Phosphate decreases urine calcium and increases calcium balance: a meta-analysis of the osteoporosis acid-ash diet hypothesis. *Nutrition Journal*.

[28] Hulley SB, Vogel JM, Donaldson CL, Bayers JH, Friedman RJ, Rosen SN (1971). The effect of supplemental oral phosphate on the bone mineral changes during prolonged bed rest. *Journal of Clinical Investigation*.

[29] Fenton TR, Lyon AW, Eliasziw M, Tough SC, Hanley DA (2009). Meta-analysis of the effect of the acid-ash hypothesis of osteoporosis on calcium balance. *Journal of Bone and Mineral Research*.

[30] Supplee JD, Duncan GE, Bruemmer B, Goldberg J, Wen Y, Henderson JA (2011). Soda intake and osteoporosis risk in postmenopausal American-Indian women. *Public Health Nutrition*.

[31] Fenton TR, Tough SC, Lyon AW, Eliasziw M, Hanley DA (2011). Causal assessment of dietary acid load and bone disease: a systematic review & meta-analysis applying Hill's epidemiologic criteria for causality. *Nutrition Journal*.

[32] Frassetto LA, Morris RC, Sebastian A (2007). Dietary sodium chloride intake independently predicts the degree of hyperchloremic metabolic acidosis in healthy humans consuming a net acid-producing diet. *American Journal of Physiology—Renal Physiology*.

[33] Frings-Meuthen P, Buehlmeier J, Baecker N (2011). High sodium chloride intake exacerbates immobilization-induced bone resorption and protein losses. *Journal of Applied Physiology*.

[34] Cappuccio FP, Meilahn E, Zmuda JM, Cauley JA (1999). High blood pressure and bone-mineral loss in elderly white women: a prospective study. *Lancet*.

[35] Devine A, Criddle RA, Dick IM, Kerr DA, Prince RL (1995). A longitudinal study of the effect of sodium and calcium intakes on regional bone density in postmenopausal women. *American Journal of Clinical Nutrition*.

[36] Morris RC, Schmidlin O, Frassetto LA, Sebastian A (2006). Relationship and interaction between sodium and potassium. *Journal of the American College of Nutrition*.

[37] Barzel US, Massey LK (1998). Excess dietary protein may can adversely affect bone. *Journal of Nutrition*.

[38] Heaney RP, Layman DK (2008). Amount and type of protein influences bone health. *American Journal of Clinical Nutrition*.

[39] Dawson-Hughes B, Harris SS, Ceglia L (2008). Alkaline diets favor lean tissue mass in older adults. *American Journal of Clinical Nutrition*.

[40] Garibotto G, Russo R, Sofia A (1996). Muscle protein turnover in chronic renal failure patients with metabolic acidosis or normal acid-base balance. *Mineral and Electrolyte Metabolism*.

[41] Caso G, Garlick PJ (2005). Control of muscle protein kinetics by acid-base balance. *Current Opinion in Clinical Nutrition and Metabolic Care*.

[42] Webster MJ, Webster MN, Crawford RE, Gladden LB (1993). Effect of sodium bicarbonate ingestion on exhaustive resistance exercise performance. *Medicine and Science in Sports and Exercise*.

[43] McSherry E, Morris RC (1978). Attainment and maintenance of normal stature with alkali therapy in infants and children with classic renal tubular acidosis. *Journal of Clinical Investigation*.

[44] Frassetto L, Morris RC, Sebastian A (1997). Potassium bicarbonate reduces urinary nitrogen excretion in postmenopausal women. *Journal of Clinical Endocrinology and Metabolism*.

[45] Wass JAH, Reddy R (2010). Growth hormone and memory. *Journal of Endocrinology*.

[46] Frassetto L, Morris RC, Sebastian A (2005). Long-term persistence of the urine calcium-lowering effect of potassium bicarbonate in postmenopausal women. *Journal of Clinical Endocrinology and Metabolism*.

[47] Vormann J, Worlitschek M, Goedecke T, Silver B (2001). Supplementation with alkaline minerals reduces symptoms in patients with chronic low back pain. *Journal of Trace Elements in Medicine and Biology*.

[48] Zofková I, Kancheva RL (1995). The relationship between magnesium and calciotropic hormones. *Magnesium Research*.

[49] Schwalfenberg G (2009). Improvement of chronic back pain or failed back surgery with vitamin D repletion: a case series. *Journal of the American Board of Family Medicine*.

[50] Groos E, Walker L, Masters JR (1986). Intravesical chemotherapy. Studies on the relationship between pH and cytotoxicity. *Cancer*.

[51] Smith SR, Martin PA, Edwards RHT (1991). Tumour pH and response to chemotherapy: an in vivo 31P magnetic resonance spectroscopy study in non-Hodgkin’s lymphoma. *British Journal of Radiology*.

[52] Raghunand N, Gillies RJ (2001). pH and chemotherapy. *Novartis Foundation Symposium*.

[53] Raghunand N, He X, Van Sluis R (1999). Enhancement of chemotherapy by manipulation of tumour pH. *British Journal of Cancer*.

